# Role of late-onset smoking in super-aged patients with diffuse large B-cell lymphoma: a real-world study in China between 2010 and 2024

**DOI:** 10.7717/peerj.21557

**Published:** 2026-07-22

**Authors:** Zhan Shi, Xi Tang, Qianwen Shen

**Affiliations:** 1 Department of Medical Oncology of Hua’Dong Hospital, Shanghai Medical College, Fudan University, Shanghai, China; 2 Department of Radiation of Hua’Dong Hospital, Shanghai Medical College, Fudan University, Shanghai, China

**Keywords:** Super-aging, Diffuse large B-cell lymphoma, Pandemic, Prognostic factors, Smoking

## Abstract

**Aim:**

To investigate the prognostic impact of early-onset smoking history (started smoking before 24 years old) on the survival of Chinese patients aged ≥ 80 years with diffuse large B-cell lymphoma (DLBCL).

**Methods:**

Patients aged ≥ 80 years DLBCL with smoke history admitted to our center from 2010 to 2024 were retrospectively enrolled. Patients who started smoking before 24 years old were defined as early-onset smoking group, while who started smoking after the age of < 24 years old were defined as late-onset smoking group. The overall survival (OS), progression-free survival (PFS) and treatment-related complications were compared between the two groups. Cox regression model was used to analyze the prognostic factors.

**Results:**

After follow-up duration of 28.7 months, 68 patients were included. The median OS of early-onset smoking group (*n* = 29) was 2.3 years, which was significantly lower than that of late-onset smoking group (*n* = 39, 3.7 years, *p* < 0.01). Multivariate analysis illuminated that late-onset smoking decreased the risk of death significantly (HR = 0.317, *p* = 0.031), as well as another risk factor: ECOG < 2 (HR = 0.569, *p* = 0.042).

**Conclusions:**

Remarkably, late-onset smoking and better performance status portended a favorable prognosis of super-aged patients with lymphoma.

## Introduction

Smoking is a definite risk factor for a variety of cancers, whereas its effect on the prognosis of lymphoma in the elderly is still unclear, especially the long-term effect of early-onset smoking. Adolescent smoking, which introduces toxins and harmful substances during this sensitive period, may cause irreversible damage to physical and mental development, leading to worse consequences. Alveolar development (ADS) is a special stage of alveolar development, which is different from adolescence and is a process of increasing and maturing alveolar number from birth to about 24 years old (Bui et al., 2018). Because of the potential for irreversible damage, smoking during ADS may not only lead to a reduction in alveolar number but also long-term abnormalities in lung structure and function (Stolz et al., 2022).

A 2010 study of the American cancer population published in Cancer found that lymphoma patients who smoked before diagnosis had a lower overall survival (Geyer et al., 2010). Most of the previous studies focused on young and middle-aged patients, and there was a lack of special analysis for the ≥80 years old population. To the best of our knowledge, there are very few retrospective studies in China that focus on the significance of early-onset smoking on the survival prognosis of super-aged patients. Through discussing the interactions between age of smoking initiation and super-aged patients with DLBCL developmental trajectories, our study aims to reveal potential adverse impact of early-onset smoking initiation patterns in the prognosis of super-aged patients with DLBCL between 2010 and 2024. Consequently, the significance of this investigation lies in its relevance to multiple interconnected clinical, research, and public health aspects.

## Materials & Methods

### Patient recruitment

The inclusion criteria were patients ≥80 years old who were diagnosed with DLBCL between July 2010 and October 2024 and had received at least four cycles of immune therapy. All participants had a history of smoking. Those with other malignant tumors or a primary central nervous system lymphoma subtype or without smoking history were excluded. The Ethics Committee of Hua’Dong Hospital Affiliated to Fudan University, Shanghai, China approval to carry out the study within its facilities (Ethical Application No. 2023K074). We received all written informed consents from each participant in our study.

Clinical follow-up ended in December 2024. The baseline data included age at Ann Arbor staging, sex, routine blood examination, B-cell immunohistochemistry (IHC) markers, smoking history, the severity of chronic obstructive pulmonary disease (COPD) evaluated according to GOLD guidelines, comorbidities, cardiac function, arterial blood gas analysis, ECOG-PS scoring and spirometry tests. The diagnostic criteria for COPD in this study was: who all had a history of tobacco exposure, cough and chest tightness, and FEV1/FVC < 0.7 after bronchodilator use (Kahnert et al., 2023). In the current study, all 39 late-onset group and 29 early-onset group met the diagnostic criteria for COPD. The Modified-Cumulative Illness Rating Scale (M-CIRS), which includes 14 systems to quantify comorbidity, was used (Gronberg et al., 2010; Ngeow et al., 2010). The new NCCN-International Prognostic Index (NCCN-IPI) (Prochazka et al., 2014) was used for patients aged >80 years in our trial. Grouping criteria: Patients who started smoking before 24 years old were defined as early-onset smoking group, while who started smoking at the age over 24 years old were defined as late-onset smoking group.

### The first-line and the second-line attenuated immune-therapy protocol

The R-Mini-CHOP regimen is the first-line immune-therapy for this population in current study. A retrospective study published in Hematologic in 2005 manifested that the addition of rituximab to reduced-dose CHOP chemotherapy appeared to be a good compromise between toxicity and efficacy in patients over 80 years of age, allowing clinicians to treat very elderly patients with curative intent (Peyrade et al., 2011b). In 2010, researchers from Tunisian published a study that also explored mini-chop in 69 people age over 70 who received six courses of mini-CEOP regimen (Laatiri et al., 2010) with the 4-year overall survival was 56%. This is also the trial foundation of chemotherapy protocol for GELA LNH 03-7B trial, and given the encouraging overall survival results of ≥80 years old lymphoma patients in the GELA LNH03-7B, the R-Mini-CHOP regimen is the first line recommended protocol for frail or super-aging patients with DLBCL in our institutional (Musolino et al., 2011; Peyrade et al., 2011b). The R-Mini-CHOP regimen includes a target-CD20 monoclonal antibody: Rituximab (375 mg/m2) on day 0, followed by Cyclophosphamide (CTX) 400 mg/m^2^/d iv on day 1, Epirubicin 35 mg/m^2^/d on day 1, Vincristine (VCR) 1 mg/d on day 1, and Methylprednisolone 50 mg/m^2^/d on days 1–5. The immunotherapy regimen was administered at 3-week intervals and at least four cycles. The reason for including elderly patients over 80 years old who completed at least four cycles of immunotherapy in this retrospective study is that the first-line induction therapy for lymphoma lasts for 4 to 6 cycles. If the number of treatment cycles completed is less than 4, it is impossible to accurately evaluate the efficacy of the first-line induction therapy. Secondly, it may cause bias and interference in the overall survival duration due to insufficient treatment duration. Additionally, this is also based on the LNH 03-7B trial by Peyrade et al. (2011a), where it was stated that: ‘All patients received six cycles of rituximab combined with low-dose CHOP (R-miniCHOP) at 3-week intervals’. The consolidation involved site radiation therapy (ISRT) was conducted with bulky (mass larger than 7.5 cm) or isolated extra-nodal mass following a complete remission (CR) or partial remission (PR) response (Knight et al., 2009).

After the failure of first-line treatment, attenuated R-Gemox (Gemcitabine+Oxaliplatin) regimen was used if the patient had good tolerance to previous treatment, or R-Bruton tyrosine kinase inhibitor (BTKi) regimen or R2 (Rituximab+Lenalidomide) regimen was used if the patient had poor tolerance.

### Evaluation of response

At the end of the therapy, computerized tomography (CT) scans or 18-fluorodeoxyglucose positron emission tomography-computed tomography (18FDG-PET/CT) were performed to evaluate the response in terms of CR, PR, stable disease (SD), or progressive disease (PD) according to the response evaluation criteria in solid tumors (Recist) 1.1 criteria (Musolino et al., 2011; Peyrade et al., 2011b). The overall response rate (ORR) was equal to the CR ratio plus the PR ratio. Progression-free survival (PFS) was defined as the time from entry onto a study until lymphoma progression or death as a result of any cause. Overall survival (OS) was defined as the period from the first date of diagnosis to the last follow-up date or death date (Cheson et al., 2007).

### Statistical analysis

All data were analyzed using SPSS (version 14.0; SPSS, Chicago, IL, USA). Survival was calculated using the Kaplan–Meier curve. Univariate analysis with the log-rank test was to evaluate various prognostic variables for OS and multivariate analysis using the Cox hazard regression model. All *p*-values are two-sided, with *p*-values ≤ 0.05 considered statistically significant. All data are shown as the hazard ratio (HR) with a 95% confidence interval (CI). In the statistical methods of this study, pairwise comparisons were conducted between late-onset smoking (exposure group) and early-onset smoking (control group), ECOG-PS score less than 2 (exposure group) *versus* 2 or higher (control group), stage I–II (exposure group) *versus* stage III–IV (control group), age under 85 years (exposure group) *versus* 85 years or older (control group), and M-CIRS score less than 7 (exposure group) *versus* 7 or higher (control group), to identify factors associated with prognosis in super-aged patients with lymphoma. The hazard ratios (HRs) compared the instantaneous risk of death between two groups (exposure group *vs.* control group) during a 2-year follow-up period. The interpretation of HR is as follows: HR = 1: No difference in risk between the two groups or the exposure group is equivalent to the control group. HR > 1: Higher risk in the exposure group, indicating a “risk factor.” HR < 1: Lower risk in the exposure group, indicating a “protective factor.”

## Results

### Clinical characteristics

**Figure 1:**
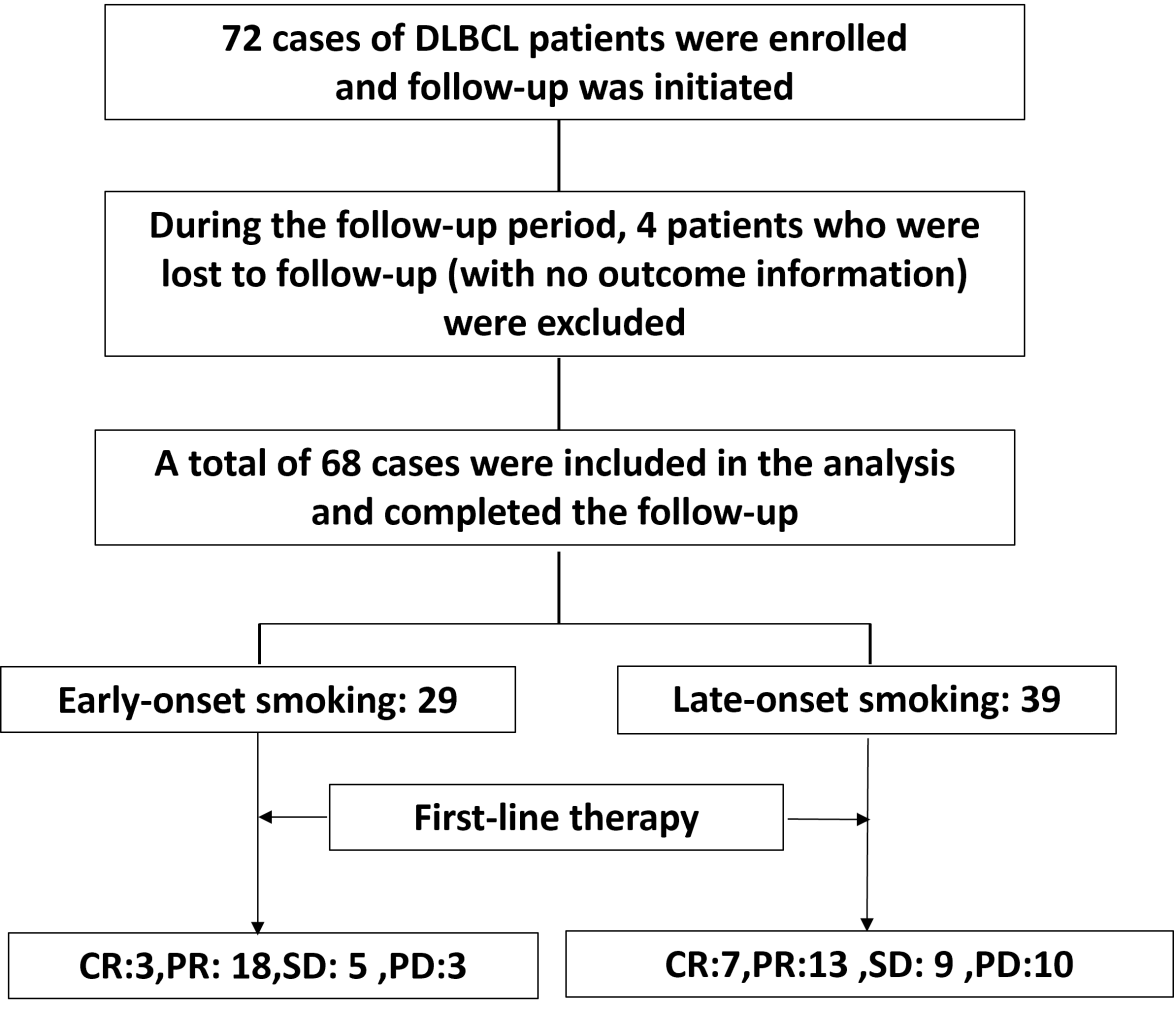
Flow chart.

For four patients censored due to loss to follow-up, all clinical baselines are summarized as the overall median age for all 68 patients was 82.5 (range: 80–92) years with 98.5% being male in Table 1. The median age of the early-onset smoking group was 82 (range : 80–90), while the median age of the late-onset smoking group was 84 (range : 80–92). The flow chart was shown as Fig. 1. There were 29 patients in the early-onset smoking group, all of whom were male, and 39 patients in the late-onset smoking group, of whom only one was female. The median age of smoking initiation in all 68 patients was 26 years old. The median smoking duration was 11.3 years. The average daily dose was 40.1 cigarettes/per day. In current study, the discovery of the severity of COPD is merely descriptive and has no causal relationship with survival. Due to the inherent limitations of observational studies, this association may be influenced by various residual confounding factors (such as smoking intensity, socioeconomic status, or unmeasured comorbidities). This finding is mainly descriptive and cannot confirm a clear causal relationship with prognosis. All cases had at least one comorbidity, with the most common being lung function decline (22%), followed by cardiopathy (18%), hypertension (13%) and type II diabetes mellitus (12%). Early-onset smoking patients were found to have poorer lung function: FEV1 measurements (L), 3.52 (3.1, 3.78) *vs.* 3.79 (3.2, 4.25) at the beginning of admission.

**Table 1:** Clinical characteristics of this study.

Characteristics	Early-onset smoking (*n* = 29)	Late-onset smoking (*n* = 39)
Gender		
Male	29	38
Female	0	1
Age, median (range)	82 (80–90)	84 (80–92)
80–84	21	24
85–89	7	12
≥ 90	1	3
Smoking index, packets/years	39 (30, 55.4)	39 (30, 59)
ECOG-PS		
0–1	16	18
2	13	21
Ann Arbor stage		
I–II	10	23
III–IV	19	16
LVEF ≤ 60%	21	20
NCCN-IPI risk group		
Low-intermediate: 2–3	3	9
High-intermediate: 4–5	10	12
High ≥ 6	16	18
M-CIRS median (range)	7 (2–16)	9 (2–18)
0–6	3	18
≥ 7	26	21
GOLD I: FEV1≥80% predicted	1	4
GOLD II: 50%≤FEV1 < 80%	4	6
GOLD III: 30%≤FEV1 < 50%	10	10
GOLD IV:FEV1 < 30%	14	19

### Toxicity of attenuated immunotherapy

The incidence of grade III-IV treatment-related complications was higher in the early-onset smoking group (32.9% *vs.* 17.9%, *p* = 0.021). The early-onset smoking group had a higher incidence of cardiovascular events than the late-onset group (12.1% *vs.* 3.2%). 21 (30.9%) patients died during the follow-up. Of these, 15 (22.1%) patients died within the first year. Among this, the number of early-onset smoking group was 10 patients, and late-onset smoking group was 5.

### Attenuated immunotherapy outcome

The median follow-up duration was 28.7 months (range: 3.4–46^+^ months). The median OS was 29.59 months (95% CI [24.39–34.78]) in this trial, with a 2-year PFS rate of 60.6% ± 6.5% and a 2-year survival rate of 61.3% ± 6.8%. The ORR of the first-line treatment response evaluation was 60.3% (CR, 10; PR, 31; SD, 14; and PD, 13). The early-onset smoking group had a lower CR rate than the late-onset group (41.6% *vs.* 58.5%, *p* = 0.03).

**Figure 2:**
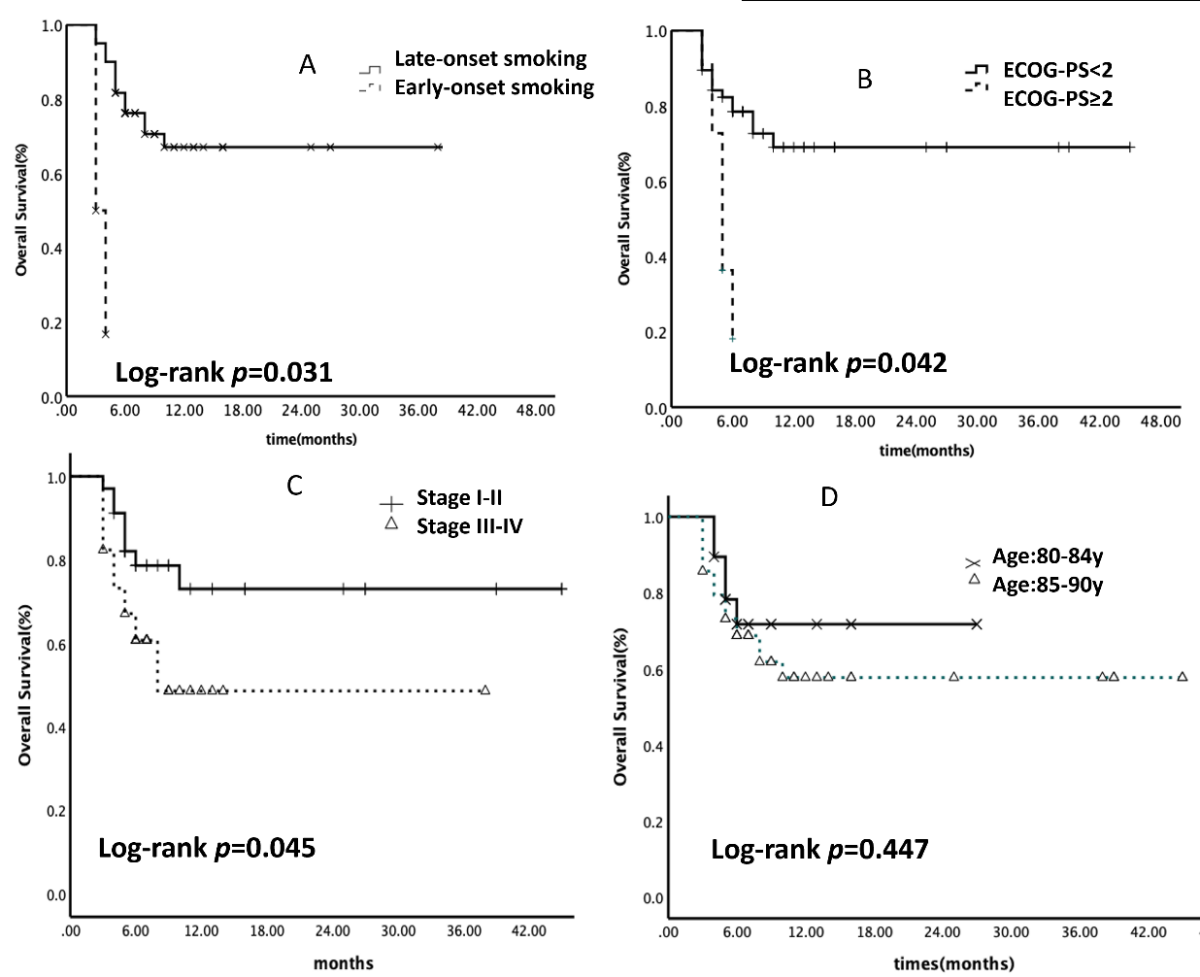
Univariate analyses and multivariate analysis results of current study. Cox regression hazards model analysis results of current study.

Table 2 and Fig. 2 presents Cox regression hazards model analysis results of current study. Univariate analyses indicated that late-onset smoking, ECOG-PS < 2, Stage I-II, age < 85 years old were four prognostic factors for a better prognostic for 2-year survival, indicating as “protective factors.” (Fig. 2; Table 2). Remarkably, based on screening from the univariate analysis, multivariate analysis illuminated that late-onset smoking decreased the risk of death significantly (HR = 0.317, *p* = 0.031), as well as another risk factor: ECOG < 2 (HR = 0.569, *p* = 0.042). Accordingly, later-onset smoking and better PS status are significantly associated with favorable prognosis in super-aged patients with lymphoma.

**Table 2:** Univariate and multivariate analysis of prognostic factors for 2-year overall survival.

Exposure group *vs.* control group	Univariate analysis		Multivariate analysis	
	2-year OS (%)	*p*-value	Hazard ratio (95% CI)	*p*-value
Late *vs.* Early-onset	76.2 *vs.* 16.7	**0.000**	0.317 (0.106–0.946)	**0.031**
ECOG-PS<2 *vs.* >=2	69.0 *vs.* 18.2	**0.001**	0.569 (0.316–0.992)	**0.042**
Stage I/II *vs.* III/IV	80.5 *vs.* 45.0	**0.045**	0.710 (0.164–3.063)	0.646
Age<85 *vs.* >=85 years	71.8 *vs.* 57.7	**0.047**	0.827 (0.319–2.140)	0.695
M-CIRS <7 *vs.* >=7	68.3 *vs.* 52.0	0.577		

**Notes.**

Data shown in bold are positive results.

## Discussion

Firstly, Shanghai is one of the cities in China that experienced the most profound aging of its population. The proportion of extremely elderly (≥80 years old) individuals has been continuously increasing, which makes research targeting this population both urgent and representative. Secondly, DLBCL is the most common type of non-Hodgkin’s lymphoma in adults, and its incidence increases significantly with age. Paradoxically, the aged ≥80 years patients have distinct characteristics because of significant decline in physiological functions: elderly patients often have multiple comorbidities (such as cardiovascular and cerebrovascular diseases, diabetes, chronic kidney disease, *etc*.), malnutrition, and frailty syndrome. Secondly, treatment tolerance is poorer especially for the aged ≥80 years old patients. More importantly, most clinical trials exclude elderly patients or those with multiple comorbidities due to safety concerns, resulting in a serious shortage of high-quality evidence-based medical evidence specifically for this specific group. Here, with a follow-up of over 14 years, we conducted a detailed analysis on the significance of early-onset smoking on the survival prognosis of super-aged patients, which is a very specific and clinically significant research topic.

Apparently, smoking is a definite risk factor for DLBCL: numerous epidemiological studies have confirmed that smoking is a definite risk factor for various cancers, including lymphoma. Smoking has also a profound impact on treatment efficacy and prognosis: studies have elucidated that smoking may affect the molecular subtypes (such as GCB/non-GCB types) and invasiveness of DLBCL by inducing gene mutations (such as TP53), epigenetic changes, and creating an immunosuppressive microenvironment. Previously, NIH investigated whether smoking prior to NHL diagnosis was associated with overall survival and conducted a meta-analysis to assess the evidence for the relationship between pre-diagnosis smoking and OS. Among 523 NHL patients, more severe smoking habits before diagnosis were associated with worse overall survival compared with never smokers (Ollberding et al., 2013). Simultaneously, early-onset smoking increases treatment complications: smoking damages the heart and lung functions, reduces the bone marrow reserve capacity, which significantly increases the risk of infection, pulmonary complications, cardiac toxicity, and bone marrow suppression in elderly patients receiving chemotherapy, potentially leading to reduced chemotherapy doses, delayed treatment courses, or premature termination, thereby affecting the therapeutic effect. Components such as polycyclic aromatic hydrocarbons in tobacco can induce the activity of the liver cytochrome P450 enzyme system, accelerate the metabolism of certain chemotherapy drugs (such as cyclophosphamide), and may result in insufficient blood drug concentration, thereby affecting the therapeutic effect. In the context of the above studies, the importance of investigating smoking history becomes evident.

Our key findings include that late-onset smoking is significantly associated with favorable prognosis in super-aged patients with lymphoma in super-aged patients with lymphoma. Since the definitions of early-onset and late-onset smoking have been clearly stated in the methods section, it is reasonable to use the time of smoking initiation as a prognostic indicator in this retrospective study with a relatively small sample size and numerous other variables. The possible mechanisms include DNA damage accumulation caused by tobacco carcinogens (such as benzene and nitrosamines), immunosuppression and decreased tolerance to treatment. Elderly patients have reduced metabolic capacity and delayed clearance of tobacco toxic substance which may aggravate organ damage Unsurprisingly the ORR of the current trial was considerably lower than that of the outcome of LNH03-7B trial (60.3% and 73%, respectively), likely because our trial population had a greater disease burden, more advanced stage. With similar median ages and regimens, the 2-year-survival in the current study was 61.3%, which was slightly higher than those of the LNH03-7B trial (59%) and a Nordic Lymphoma Group study (49%) (Wasterlid et al., 2021). The reason for this could be that the ECOG-PS of the current cohort (no ECOG-PS score 3–5 in our study) was better than that of the LNH 03-7B group (ECOG-PS score 4–5 accounted for 40%), suggesting better treatment tolerability and recovery capacity.

Previous trials revealed that age and poor PS status have adverse effects on survival (Shi et al., 2019; Shi et al., 2016). Similarly, in the current trial, ECOG-PS≥2 had adverse prognostic effect on survival in univariate and multivariate analysis. The clinical implications were smoking history should be systematically evaluated at the time of initial diagnosis. Unfortunately, the limitations of this study include selection bias in a real-world retrospective study, and since current study only included patients who had completed at least four treatment cycles, there may be potential selection bias; failure to quantify the dose–response relationship of smoking; the sample size was not big enough and lack of exploration of molecular mechanisms (*e.g.*, TP53 mutations and epigenetic alterations).

Regarding the combination of the labels “China Shanghai”, “super-aged”, and “DLBCL”, there is almost no research conducted internationally. Exploring the interaction between smoking and the prognosis of the Chinese people with DLBCL can generate local data with Chinese characteristics. This can produce more persuasive local evidence to promote local smoking control policies and health aging publicity in Shanghai. Social initiatives to ban smoking or delay the age of smoking initiation are urgently needed, especially for adults under the age of 24.

## Conclusions

Importantly, we revealed that early-onset smoking was associated with poorer outcomes, whereas late-onset smoking and better performance status portended more favorable survival of super-aged lymphoma patients.

##  Supplemental Information

10.7717/peerj.21557/supp-1 Supplemental Information 1 Raw data

10.7717/peerj.21557/supp-2 Supplemental Information 2 Codebook for raw data

## Additional Information and Declarations

### Data Availability

The following information was supplied regarding data availability: The raw data is available in the Supplemental Files.
