# Supplementary material for: Role of late-onset smoking in super-aged patients with diffuse large B-cell lymphoma: a real-world study in China between 2010 and 2024
Source: PeerJ. 2026 Jul 22;14:e21557. doi: 10.7717/peerj.21557 (PMC13401357; doi:10.7717/peerj.21557)
Supplement: Supplemental Information 2 [file peerj-14-21557-s002.docx]

ECOG-PS=2:1; ECOG-PS<2:0

Age≥84yrs:1; Age<84yrs:0

III or IV stage:1; I or II stage:0

Albumin< 30g/L:1; Albumin>=30:0

BMI<20:1; BMI>=20:0

Anemia:1; non-anemia:0

M-CIRS≥7:1; M-CIRS<7:0

die1; live:0

early smoking:1; late smoking:0

DEL:1; non-DEL:0
